# Physiological and Proteomic Analysis of Sorghum Bicolor Seedling Leaves Reveals Molecular Responses to PEG-Induced Drought Stress

**DOI:** 10.3390/plants15081255

**Published:** 2026-04-18

**Authors:** Hongbing Li, Qilong Han, Zhao Yang, Meijing Cheng, Qingbo Ke, Sang-Soo Kwak, Xiping Deng, Suiqi Zhang

**Affiliations:** 1 State Key Laboratory of Soil and Water Conservation and Desertification Control, College of Soil and Water Conservation Science and Engineering, Northwest A&F University, Yangling 712100, China; 2 Plant Systems Engineering Research Center, Korea Research Institute of Bioscience and Biotechnology (KRIBB), Daejeon 34141, Republic of Korea

**Keywords:** drought stress, sorghum bicolor, proteomics, physiological adaptation, osmotic stress

## Abstract

Drought stress significantly constrains crop productivity and yield stability. Sorghum (*Sorghum bicolor* L. Moench), a C4 cereal widely cultivated in arid and semi-arid regions, exhibits high water-use efficiency and remarkable drought tolerance. Understanding both the impacts of drought and the plant’s response mechanisms is essential for enhancing drought resilience in this crop. In this study, physiological changes and differential protein accumulation were analyzed in leaves of the sorghum inbred line BT × 623 under 10% PEG-6000-induced drought stress. The physiological adaptation to drought was characterized by improved water retention and mitigation of oxidative damage through the synergistic action of antioxidant enzymes. Using two-dimensional electrophoresis (2-DE) and MALDI-TOF-TOF mass spectrometry, 43 protein spots were successfully identified, corresponding to 38 unique proteins differentially expressed under osmotic stress. These proteins function in diverse biological processes, including protein synthesis, processing, and degradation; photosynthesis; carbohydrate and energy metabolism; transcriptional regulation; stress and defense; lipid and membrane metabolism; and amino acid metabolism. Proteomic profiling revealed that the coordinated modulation of multiple functional groups, such as those involved in photosynthesis, energy metabolism, transcriptional adjustment, ROS scavenging, and protein turnover, underpins sorghum’s osmotic stress adaptation. These findings provide key insights into the drought resistance mechanisms of sorghum at both physiological and proteomic levels.

## 1. Introduction

Drought stress is regarded as one of the principal constraints on plant growth and crop productivity in most agricultural regions worldwide [1,2]. Given the long-term effects of global warming, drought events are anticipated to occur with greater frequency in the future [3]. To develop food crops with enhanced productivity in marginal lands, a comprehensive understanding of the intrinsic mechanisms underlying plant stress responses is imperative. The mechanisms by which plants respond to drought stress are highly complex, involving the regulation of a series of changes at morphological, physiological, and molecular levels [4,5].

Sorghum (*Sorghum bicolor* (L.) Moench) exhibits remarkable adaptation to arid and adverse environments [6], positioning it as an increasingly vital food source in semi-arid and arid regions. As a valuable germplasm resource, sorghum serves as a promising model system for elucidating the molecular mechanisms underlying drought resistance in cereal crops. Its advantageous traits include high photosynthetic efficiency, a short growth cycle, low water requirements, and pronounced drought tolerance [7]. These characteristics render it particularly suitable for cultivation in marginal soils and water-limited regions. Unraveling the molecular basis and inherent regulatory mechanisms of drought tolerance in sorghum would facilitate the exploration of genetic resources for crop improvement.

Under drought conditions, plants typically accumulate reactive oxygen species (ROS), leading to oxidative stress. Excessive ROS production can inflict cellular damage through oxidation of lipids, proteins, and nucleic acids, ultimately resulting in cell injury and death [8]. To maintain ROS homeostasis, plants employ enzymatic antioxidant systems-including superoxide dismutase (SOD), peroxidase (POD), ascorbate peroxidase (APX), catalase (CAT), polyphenol oxidase (PPO), and glutathione reductase (GR)—to mitigate oxidative damage [8,9,10,11]. The capacity to minimize ROS accumulation and mitigate their detrimental effects is closely associated with plant drought tolerance [12].

Although sorghum’s drought resistance has been largely attributed to morphological and physiological features—such as C_4_ photosynthesis, a dense root system [6,13], high leaf epicuticular wax deposition [14], sustained stomatal opening and photosynthesis under low water potential, efficient soil water extraction [15], and osmotic adjustment ability [16]—the responses of antioxidant enzymes in leaves under osmotic stress remain inadequately characterized, particularly their dynamic changes across different time points following stress exposure. Therefore, investigating the physiological and molecular mechanisms governing drought resistance in sorghum will provide critical insights for developing effective genetic enhancement strategies.

Given the significance of sorghum stress resistance, its molecular mechanisms have been extensively investigated. Transcriptomic profiles of sorghum under osmotic stress, high sodium chloride, abscisic acid (ABA), heat, and drought treatments have been systematically characterized [17,18,19]. Recently, Abdel-Ghany (2020) conducted a transcriptome analysis of drought-resistant and drought-sensitive sorghum seedlings subjected to PEG-induced drought stress, identifying a suite of drought-responsive genes, including many encoding uncharacterized proteins associated with drought resistance [20]. These studies provide preliminary molecular insights into drought resistance in sorghum and yield valuable transcriptomic data regarding its response to water deficit. While they offer important directions for future research on drought tolerance mechanisms, the fundamental intrinsic mechanisms underlying sorghum’s drought resistance remain to be fully elucidated.

The overall function of a gene within an organism is ultimately realized through its encoded protein at the cellular level. Transcriptional-level gene expression information alone is often insufficient to fully elucidate the precise roles of genes in cellular processes, given that biological outcomes in cells, tissues, and organs are primarily governed by proteins rather than genes. Merely observing changes in expression levels through gene or RNA sequencing may not capture the entirety of cellular regulatory processes and their consequent biological effects. Moreover, the expression levels of genes do not strictly correlate with the abundance of their corresponding proteins [21]. Therefore, investigating the molecular mechanisms of sorghum’s response to drought stress from a proteomic perspective, along with exploiting drought resistance genetic resources and identifying as well as cloning drought-resistant proteins, holds significant research value for clarifying the molecular basis of drought tolerance in sorghum. Such efforts are particularly crucial for the rational utilization of drought-resistant traits. Furthermore, this research bears substantial theoretical and practical importance for enhancing drought resistance and improving water-use efficiency in crops such as wheat and maize.

With the completion of genomic sequencing for an increasing number of organisms—including sorghum [22], whose BT × 623 line, with a genome size of 750 Mb, positions it as an ideal model crop—proteomics has emerged as a crucial methodology in the post-genomic era. Sorghum exhibits high water-use efficiency, primarily utilizing soluble sugars and proteins as key osmoregulatory substances under drought stress [13]. Following severe drought, sorghum demonstrates a rapid recovery capacity and can achieve substantial yield once water becomes available. In response to water deficit, sorghum undergoes a series of adaptations spanning cellular to physiological levels, involving significant changes in the abundance and composition of numerous proteins. Proteomic analyses will facilitate a deeper understanding of the protective and adaptive mechanisms that enable sorghum to withstand drought stress.

The functional identification of numerous drought-responsive proteins has been reported in model species such as *Arabidopsis* [23,24], rice [25], maize [26,27], and other plants [28,29,30,31,32]. These studies have substantially elucidated the molecular mechanisms underlying plant responses to water deficit and demonstrate the broad application potential of proteomics in research on plant drought resistance. However, functional insights remain limited in sorghum to date. Proteomic analyses of two *Sorghum bicolor* landraces—drought-resistant (accession number 11434) and drought-sensitive (accession number 11431)-under drought stress revealed altered protein expression profiles in leaves. The most notable differences between resistant and sensitive genotypes involved proteins associated with energy balance, metabolic processes, and molecular chaperones [33]. Additionally, physiological and comparative proteomic studies in sorghum have identified protein groups responsive to abiotic stresses including salinity, cadmium/copper exposure, and drought conditions [34,35,36,37,38,39,40,41,42].

Although numerous proteomic studies have investigated drought stress responses in sorghum, the mechanism by which leaves—the primary vegetative organs—respond to drought at the protein level remains unclear. Unlike previous sorghum proteomic studies that often compared different cultivars or examined later stress responses, this study focuses on the proteome dynamics of the reference genotype BT × 623 under PEG-induced osmotic stress at a time point representing established physiological adjustment (24 h), rather than early signaling. By coupling this single-time-point proteomic analysis with time-resolved physiological measurements across multiple early time points (3, 6, and 9 h), we establish direct links between early physiological adjustments and specific protein changes at the stabilized phase, providing an integrated view of the drought response mechanisms during the transition from early sensing to established adaptation. Moreover, comparison with existing transcriptomic datasets reveals discrepancies between transcript and protein abundances, highlighting potential post-transcriptional regulatory events critical for drought adaptation in sorghum. A comprehensive understanding spanning from physiological to molecular traits is essential to identify key genes involved in sorghum’s drought resilience. Thus, to further elucidate the molecular events underlying sorghum’s response to water deficit, physiological assessments and proteomic analyses were employed to compare physiological changes and differentially accumulated proteins in the sorghum inbred line BT × 623 under PEG-induced drought stress. This study aims to uncover sorghum’s drought resistance adaptation mechanisms, identify drought-responsive proteins, determine major protein categories involved in the stress response, and screen key drought-related protein genes for potential use in genetic engineering to enhance drought tolerance in cereal crops. The findings are expected to provide deeper insights into the mechanisms of drought resistance in sorghum, thereby supporting efforts to improve the plant’s drought adaptability.

## 2. Materials and Methods

### 2.1. Seedling Cultivation and Polyethylene Glycol (PEG) Treatment

Surface-sterilized seeds of the Sorghum bicolor genotype BTx623, whose genome sequence has been published (http://www.phytozome.net/sorghum, accessed on 22 June 2021), were germinated for 4 days at 25 °C. After germination, healthy seedlings were transferred to a plastic container with 4 L of half-strength Hoagland nutrient solution [composed of Ca(NO_3_)_2_, KNO_3_, MgSO_4_, (NH_4_)_3_PO_4_, iron salt solution, and trace elements; pH = 6.0]. The nutrient solution was replaced with full-strength Hoagland solution 6 days after transplanting. Continuous aeration was provided, and the pH was maintained at 6.0 using 0.1 M HCl or 1 N KOH.

The cultivation of sorghum seedlings in this experiment was conducted in an artificial climate chamber under the following conditions: light period of 14 h, light intensity of 450 μmol m^−2^ s^−1^, temperature of 28 °C, nighttime temperature of 23 °C, and humidity of 40–50%.

To induce drought stress, 10% PEG-6000 (−0.2 MPa) was applied at 8:00 a.m., 12 days after transplanting. Samples were collected after 3, 6, 9, and 24 h of treatment for analysis. Untreated plants served as controls. For physiological assays, each treatment included three biological replicates, with two technical replicates per biological replicate. For proteomic analysis, three independent biological replicates were used.

### 2.2. Leaf Relative Water Content (RWC) and Leaf Water Potential Measurement

To assess the water status under PEG-induced drought stress, the relative water content (RWC) was measured according to the method of Ahmad et al. [43]. In brief, the uppermost fully expanded leaves were weighed to obtain fresh weight (FW), saturated in water for 6 h at 20 °C under light conditions, and then weighed again to determine turgid weight (TW). The samples were subsequently oven-dried at 80 °C for 48 h to obtain dry weight (DW). RWC was calculated using the formula: RWC = (FW − DW)/(TW − DW) × 100. Prior to excision, the uppermost fully expanded leaves were covered with aluminum foil, and water potential was measured using a pressure chamber (Model 3500, Soil Moisture Equipment Corp., Santa Barbara, CA, USA). Each treatment consisted of six replicates.

### 2.3. MDA Contents Measurement

Lipid peroxidation was assessed by measuring malondialdehyde (MDA) content using a modified thiobarbituric acid (TBA) method [44]. Approximately 0.1 g of leaf tissue was homogenized in 1.0 mL of 10% trichloroacetic acid (TCA) using a mortar and pestle. The homogenate was centrifuged at 10,000 rpm for 20 min. A reaction mixture consisting of 0.5 mL of the supernatant and 1 mL of TBA solution was heated at 100 °C for 30 min, rapidly cooled on ice, and centrifuged again at 10,000 rpm for 20 min. Absorbance was measured at 450 nm, 532 nm, and 600 nm using a UV spectrophotometer (Shimadzu UV-2550, Kyoto, Japan). Three biological replicates were performed.

### 2.4. Antioxidative Enzyme Activity Assays

To analyze the activities of SOD, POD, and PPO, total soluble protein was extracted from the third leaf from the top of sorghum plants subjected to 10% PEG-6000 treatment. Approximately 0.1 g of frozen leaf tissue was homogenized in ice-cold 50 mM potassium phosphate buffer (pH 7.0) containing 1 mM EDTA-Na_2_ and 2% (*w*/*v*) polyvinylpolypyrrolidone (PVPP) using a pre-chilled mortar and pestle. The homogenate was centrifuged at 15,000× *g* for 20 min at 4 °C, and the supernatant was immediately used for enzyme assays.

Protein concentration was determined using the Bradford method [45] with a Bio-Rad (Hercules, CA, USA) protein assay kit, using bovine serum albumin as the standard. SOD activity was measured based on the inhibition of the photochemical reduction of nitro blue tetrazolium (NBT) at 560 nm [46]. POD activity was assayed according to Kwak et al. [47] using pyrogallol as the substrate. One unit of POD activity was defined as the amount of enzyme required to produce 1 mg of purpurogallin from pyrogallol in 20 s, measured at 420 nm.

PPO activity was determined by monitoring the increase in absorbance at 420 nm at 15 s intervals for 5 min. The reaction mixture consisted of 0.05 mL of enzyme extract, 0.4 mL of 100 mM catechol, and 0.8 mL of 0.1 M phosphate buffer (pH 5.0) at 25 °C. A control without enzyme extract and a blank with buffer were included. Enzyme activity was calculated from the linear region of the absorbance curve, with one unit defined as an increase of 0.01 in absorbance per minute.

### 2.5. Measurement of Free Proline Content

The free proline content in drought-treated plants was determined spectrophotometrically following the method of Bates et al. (1973) [48]. In brief, 0.1 g of leaf tissue was homogenized in 3 mL of 3% sulfosalicylic acid and centrifuged at 10,000× *g* for 15 min. Subsequently, 1 mL of the supernatant was combined with 1 mL of glacial acetic acid and 1 mL of ninhydrin reagent in a test tube. The mixture was incubated in a boiling water bath at 100 °C for 30 min. After cooling, 2 mL of toluene was added, and the solution was vortexed for 30 s. The absorbance of the upper toluene phase (containing proline) was measured at 520 nm using a UV-2550 spectrophotometer (Shimadzu, Kyoto, Japan), with toluene serving as the blank. Proline content (μg/g fresh weight) was quantified using a standard curve prepared with known proline concentrations according to the ninhydrin acid reagent method [48].

### 2.6. Protein Extraction and Quantification

Freshly harvested sorghum leaves were ground using a mortar and pestle pre-cooled with liquid nitrogen. Total proteins were extracted from the powdered plant tissue using the trichloroacetic acid (TCA)–acetone precipitation method [49]. Protein concentration was determined using the Bradford reagent [45].

### 2.7. Isoelectric Focusing (IEF) and SDS–PAGE Conditions

For each sample, 500 μg of protein was combined with rehydration buffer (7 M urea, 2 M thiourea, 4% *w*/*v* CHAPS, 65 mM DTT, 0.2% Bio-Lyte, and 0.001% *w*/*v* bromophenol blue) to a final volume of 330 μL. Protein samples were loaded onto 17 cm pH 4–7 Bio-Rad ReadyStrip™ IPG strips (Bio-Rad, Beijing, China) according to the manufacturer’s protocol. Isoelectric focusing (IEF) was performed using a PROTEAN IEF system (Bio-Rad) under the following conditions: 250 V for 1 h, 500 V for 1 h, 1000 V for 1 h, a linear gradient from 1000 V to 10,000 V over 5 h, 10,000 V for 6.5 h, and a final hold at 500 V. After IEF, the strips were incubated for 15 min in equilibration buffer I (6 M urea, 2% SDS, 0.375 M Tris-HCl, pH 8.8, 20% glycerol, 2% DTT), followed by 15 min in equilibration buffer II (6 M urea, 2% SDS, 0.375 M Tris-HCl, pH 8.8, 20% glycerol, 2.5% iodoacetamide). The equilibrated strips were then transferred onto 12% polyacrylamide gels-selected after comparative testing of 10%, 12%, and 15% concentrations for optimal resolution-and sealed with 1% agarose. Electrophoresis was carried out using a PROTEAN II XL system (Bio-Rad), beginning at 1 W per gel for 45 min, then increasing to 12 W per gel for 6 h.

### 2.8. Staining of 2-DE Gels and Gel Images Analysis

Protein spots were visualized using colloidal Coomassie Brilliant Blue G-250 staining. Each treatment was analyzed in three biological replicates, and gels were scanned with a UMAX PowerLook scanner (Bio-Rad). Protein spot quantification was performed with PDQuest 8.0 software (Bio-Rad), using the “total density in gel image” method to normalize spot intensities across gels. Over-saturated spots were omitted from analysis. Statistical significance between treated samples and controls was assessed by Student’s *t*-test (*p* < 0.05). Only protein spots exhibiting statistically significant intensity differences were selected for further analysis.

### 2.9. MALDI-TOF-TOF MS and Database Query

Protein spots exhibiting a ≥2-fold change with a quality score ≥80 (*p* < 0.05) were excised from two-dimensional gels. The excised gel plugs were destained, dried at 50 °C, digested with trypsin, and extracted with 50% acetonitrile [50]. Tryptic peptides were analyzed using a REFLEX MALDI-TOF-TOF mass spectrometer (Bruker Daltonics, Bremen, Germany) operated in positive ion reflector mode. Mass spectra were calibrated using trypsin autolysis peaks as internal standards. Protein identification was performed by searching the NCBI non-redundant protein sequence database using the Mascot software (http://www.matrixscience.com, accessed on 8 April 2026) [51]. Identifications were considered confident with Mascot scores exceeding 73 (corresponding to *p* < 0.05). Gene locus, protein function, and subcellular localization were annotated using Phytozome v11.0 with the *Sorghum bicolor* v3.1 genome annotation database (https://phytozome.jgi.doe.gov, accessed on 8 April 2026), along with the SIB bioinformatics resource portal (http://www.expasy.org, accessed on 8 April 2026) and UniProtKB complete proteome database (http://www.uniprot.org, accessed on 8 April 2026).

### 2.10. Functional Categorization and Subcellular Localization of Detected Proteins

The identified proteins were functionally classified using Gene Ontology (GO) (http://geneontology.org/, accessed on 8 April 2026) based on the sorghum and maize genome annotation databases. Subcellular localization of proteins was predicted using the SIB Bioinformatic Resource Portal (http://www.expasy.org/proteomics, accessed on 9 August 2024) and the UniProtKB Complete Proteome database (http://www.uniprot.org, accessed on 8 April 2026). Gene Ontology terms were assigned to all 43 protein spots manually according to their biological processes and cellular components.

### 2.11. Statistical Analysis

Statistical analysis was performed using the Statistical Package for the Social Sciences (SPSS version 17.0 for Windows; SPSS Inc., Chicago, IL, USA). Data were subjected to one-way analysis of variance (ANOVA), and mean differences were compared using the Tukey–Kramer test (*p* < 0.05). Multiple comparisons were further examined with the least significant difference (LSD) test. All experiments included at least three independent replicates. Figures were generated using SigmaPlot 12.0, and statistically significant differences (*p* < 0.05) are indicated by different letters in the figures.

## 3. Results

### 3.1. Effect of Drought Stress on Leaf Relative Water Content (RWC) and Leaf Water Potential

**Figure 1:**
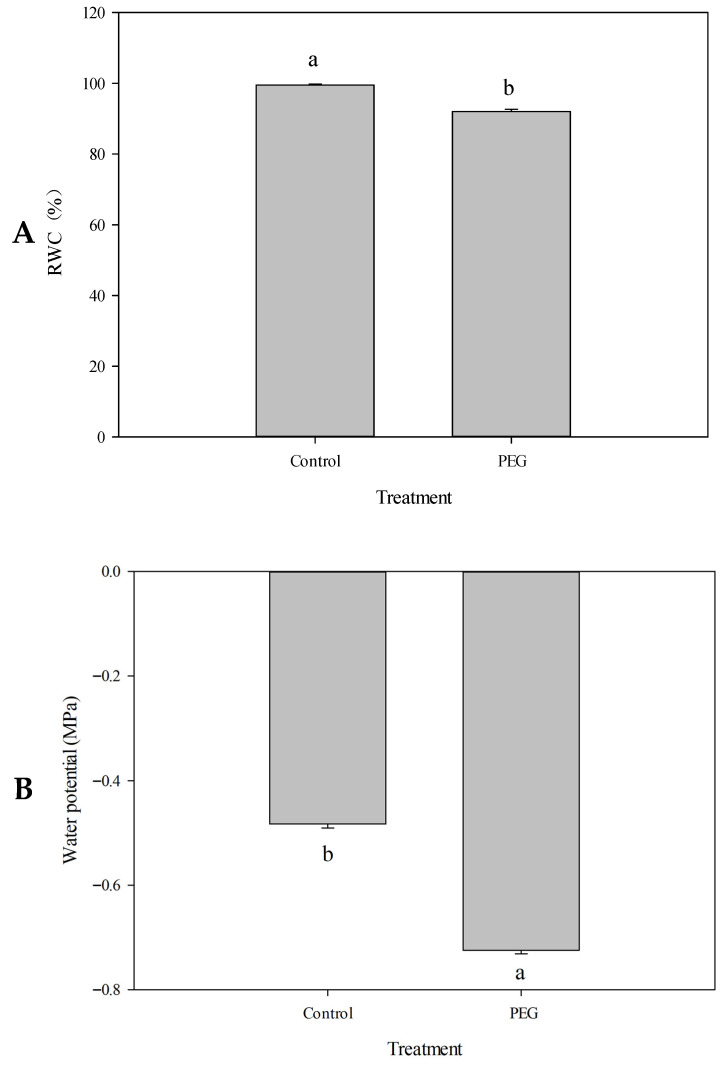
Effects of imitation drought stress on Relative Water Content (RWC) and leaf water potential of sorghum. Changes in RWC (**A**) and Water potential (**B**) of sorghum leaf in response to imitated drought stress (two-week-old seedlings treated with 10% PEG-6000 for 3 h). Data presented mean ± SD (*n* = 3). Different letters indicate statistically significant differences at *p* < 0.05.

To determine whether PEG-induced drought stress affects sorghum growth, leaf relative water content (RWC) and leaf water potential were measured. As shown in Figure 1, under well-watered conditions, the leaf RWC of sorghum seedlings was nearly 100%, and the leaf water potential was approximately −0.5 MPa. After 3 h of PEG-induced osmotic stress, leaf RWC decreased to 92%, and leaf water potential declined to −0.72 MPa. In the early stage of PEG-simulated drought, PEG-6000 primarily induced osmotic stress, leading to water loss in leaf cells and consequent reductions in RWC and leaf water potential. These changes triggered stomatal closure, thereby limiting the leaf transpiration rate. Ultimately, the reduction in water loss assisted the sorghum plants in maintaining favorable water status and enhancing drought resistance. These results suggest that leaf RWC and water potential can serve as reliable indicators of plant water status, and the capacity to maintain adequate water homeostasis reflects the drought adaptability of sorghum.

### 3.2. Drought Stress Induced MDA and Proline Accumulation in Leaves of Sorghum

**Figure 2:**
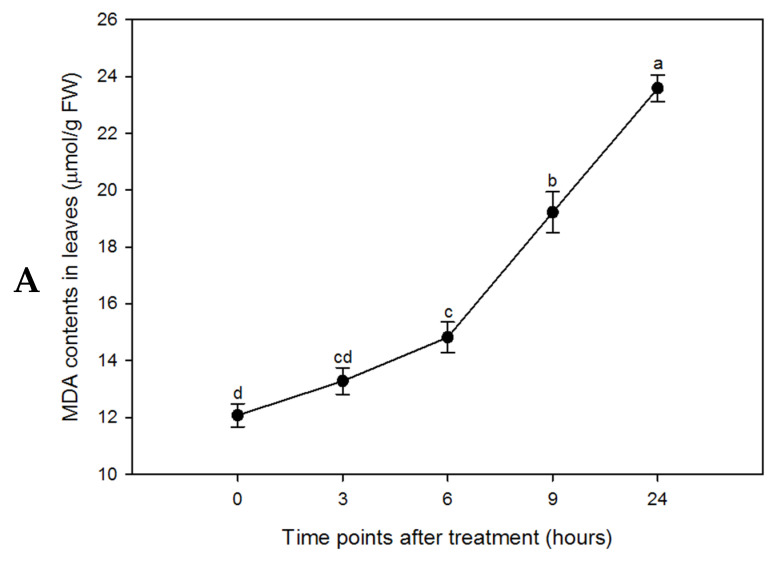

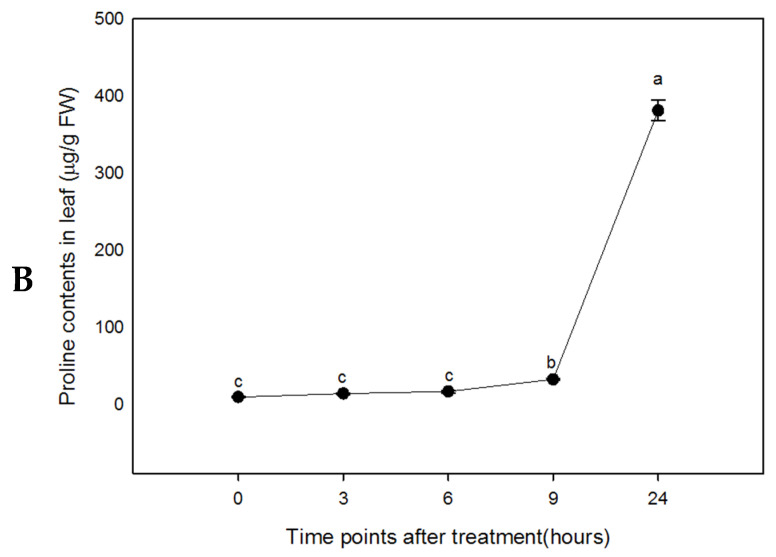
MDA and Proline analysis for determination of lipid peroxidation in sorghum leaves under imitation drought stress. Changes in MDA (**A**) and Proline (**B**) contents of sorghum leaf in response to PEG simulated drought stress (two-week-old seedlings treated with 10% PEG-6000). Data presented mean ± SD (*n* = 3). Different letters indicate statistically significant differences at *p* < 0.05.

We next assessed the effects of PEG-induced drought stress on the accumulation of malondialdehyde (MDA) and proline in sorghum leaves. MDA, a key product of polyunsaturated fatty acid peroxidation, serves as an indicator of lipid peroxidation and a biomarker of oxidative stress. As shown in Figure 2, prior to stress, MDA and proline contents were 12 μmol/g FW and 9.31 μg/g FW, respectively. With prolonged stress, both metabolites increased significantly, reaching 23.59 μmol/g FW and 375 μg/g FW, respectively, at 24 h after treatment, indicating drought-induced accumulation of MDA and proline. The accumulation of osmoprotectants such as proline under stress represents a primary defense mechanism to sustain cellular osmotic balance [52,53]. The ability to accumulate proline has been linked to enhanced stress tolerance in various plant species, including sorghum [37,38,54,55]. Stress-induced proline accumulation may contribute to antioxidative defense by scavenging free radicals or activating antioxidant systems, thereby mitigating oxidative damage [56,57]. MDA content in sorghum leaves exhibited a similar trend during drought stress: a gradual increase during the first 9 h, followed by a sharp rise from 9 to 24 h. The marked accumulation of proline likely helps alleviate MDA-induced oxidative damage and supports the activation of antioxidant mechanisms.

### 3.3. Drought Stress Induced the Increase in Antioxidant Enzyme Activity in Leaves

**Figure 3:**
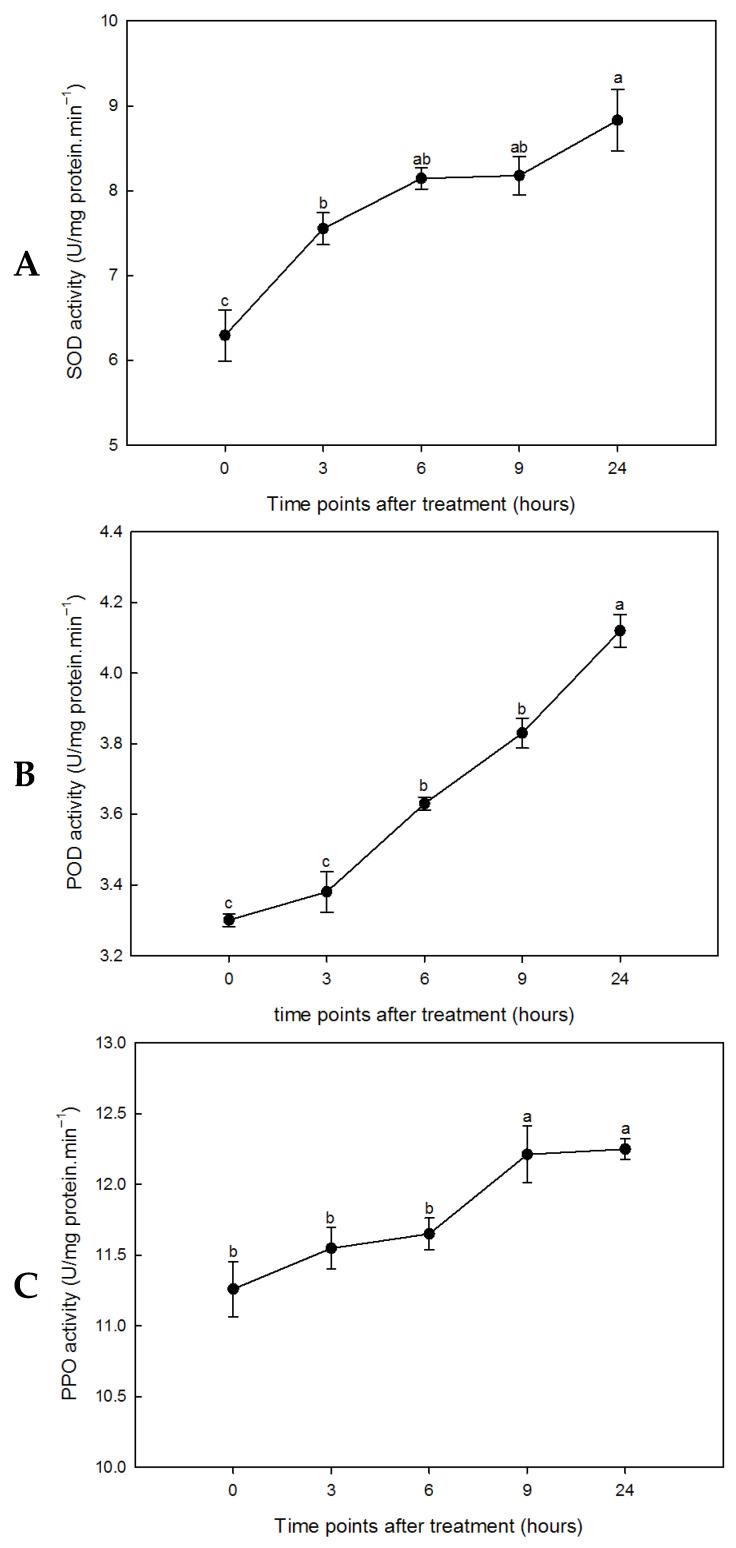
Antioxidative enzymes ((**A**): SOD; (**B**): POD; (**C**): PPO) activity of sorghum leaf in response to simulated drought stress (two-week-old seedlings treated with 10% PEG-6000). Data presented mean ± SD (*n* = 3). Different letters indicate statistically significant differences at *p* < 0.05.

Reactive oxygen species (ROS)—including superoxide radical anions, hydrogen peroxide, and hydroxyl radicals—are highly reactive and toxic molecules that can induce oxidative damage and cell death. To examine whether drought resistance in sorghum involves enhanced ROS scavenging capacity, we measured the activities of peroxidase (POD), superoxide dismutase (SOD), and polyphenol oxidase (PPO) at various time points following drought stress (Figure 3). All three enzymes showed significantly increased activities over the course of the stress treatment. Analysis of antioxidant enzyme dynamics revealed similar trends in sorghum leaves throughout the stress period. Plants possess an intrinsic protective system mediated by antioxidant enzymes, which functions to mitigate ROS-induced damage and maintain normal cellular function [58]. The balance between ROS production and antioxidant enzyme activity determines the extent of oxidative signaling and/or damage [59]. Sustaining high levels of antioxidative enzyme activities may enhance drought tolerance by improving the capacity to counteract oxidative injury [60]. Our results demonstrate that SOD, POD, and PPO activities in sorghum leaves increased gradually during the early phase of drought stress. These findings support the view that efficient antioxidative properties contribute to improved protection against oxidative stress in leaves under water-deficient conditions.

### 3.4. Changes in Proteomic Expression Patterns in Sorghum Seedling Leaves in Response to PEG Imitation Drought Stress

**Figure 4:**
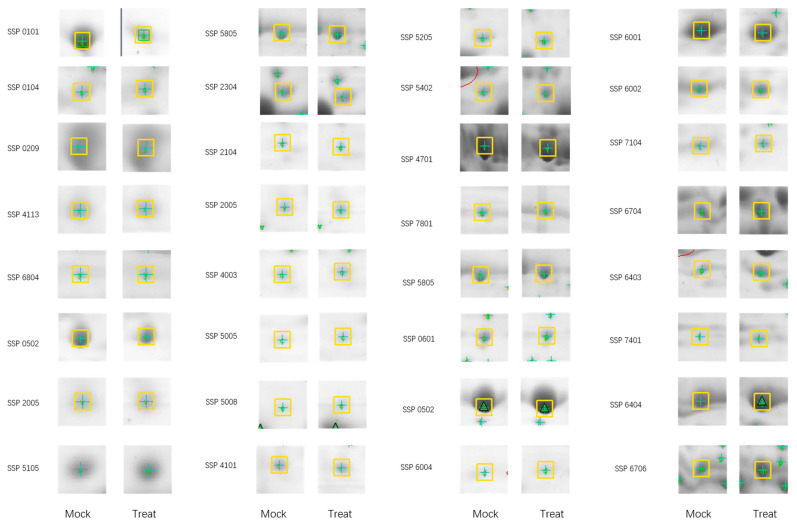
Two-D gel analysis with proteins isolated from the leaves of control sorghum seedlings (Mock) or the leaves of PEG-6000 treated sorghum seedlings (Treat) and harvested at 24 h post treatment. A comparison of 40 randomly selected protein spots is illustrated in Figure 4. The complete set of protein spot comparisons is presented in Supplementary Figure S1. Protein spot (Automatic allocation of protein serial number by PDQuest Software) with altered expression levels (fold change > 2.0, quality score >80, *p* < 0.05) and were selected for MALDI-TOF-TOF analysis.

Two-week-old sorghum seedlings were subjected to 10% PEG-6000 treatment for 24 h. Proteins extracted from the leaves were separated by two-dimensional electrophoresis (2-DE) and visualized with colloidal Coomassie Brilliant Blue staining (Figure 4). A total of 708 protein spots were reproducibly detected across gels. Among these, 387 spots were either not consistent across replicates or were exclusively present in the control samples (Supplementary Figure S1). Of the remaining 321 spots, 71 (22.1%) were up-regulated and 51 (15.9%) were down-regulated in PEG-6000 treated samples compared with the untreated control (Table 1). From these differentially expressed proteins, 36 up-regulated and 18 down-regulated spots—exhibiting at least a 2.0-fold change and a quality score above 80—were selected for identification by MALDI-TOF-TOF mass spectrometry (Table 2 and Table 3).

### 3.5. Identification of Drought-Responsive Proteins by MALDI-TOF-TOF MS Analysis

MALDI-TOF-TOF mass spectrometric analysis of the selected protein spots, followed by peptide mass fingerprint (PMF)-based MASCOT database searching, successfully identified 43 out of 54 spots (Table 4). Discrepancies between the theoretical and experimental molecular weight (MW) and isoelectric point (pI) were observed for some proteins. Such discrepancies are common in 2-DE experiments, often arising from the presence of highly similar isoforms, proteolytic cleavage, co-/post-translational modifications (PTMs), or artificial modifications. Importantly, these inconsistencies did not compromise the identification of the majority of proteins, which provided valuable insights into the molecular mechanisms underlying the sorghum response to PEG-induced drought stress.

### 3.6. Functional Classification and Subcellular Location of Identified Proteins

To better understand the characteristics of drought-resistant plants and the functional roles of proteins involved in drought stress responses, the identified proteins were functionally classified using genome annotation databases and the Complete Proteome database. Gene Ontology terms were assigned to all 43 protein spots for specific functional groups manually according to their biological processes and cellular components (Table 5).

**Figure 5:**
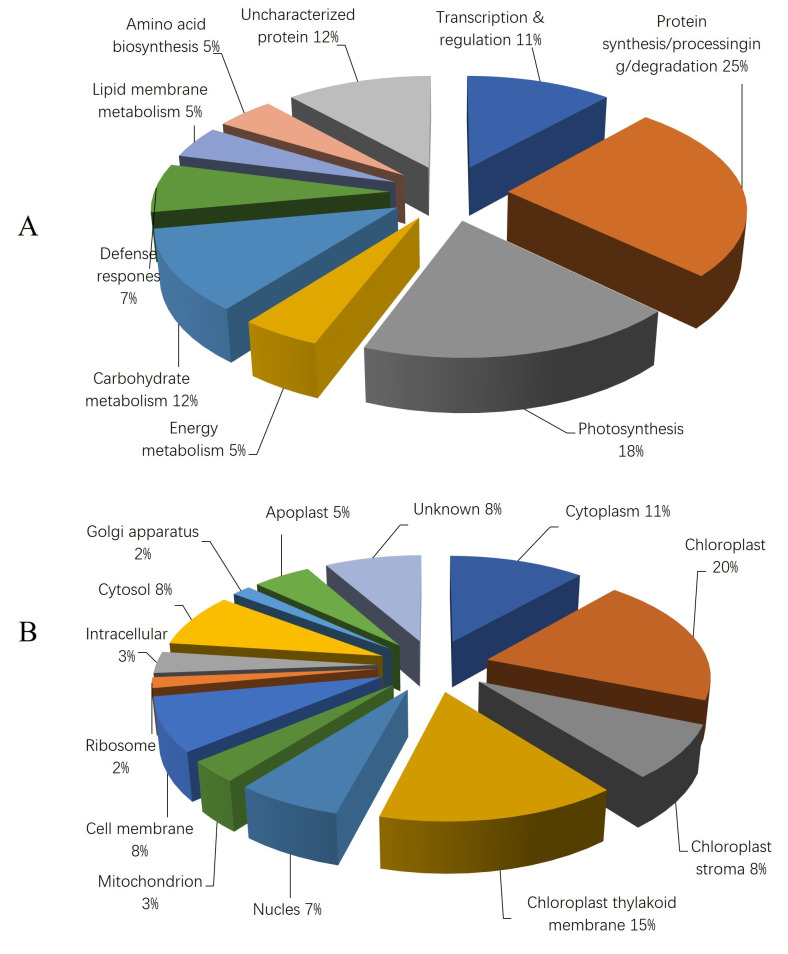
The frequency distribution for the 43 identified proteins in the leaves of sorghum seedlings within functional categories determined based on their biological functions (**A**) and subcellular localization (**B**).

Based on biological function, the identified proteins were classified into nine categories: transcription and regulation; protein synthesis, processing, and degradation; photosynthesis; energy metabolism; carbohydrate metabolism; stress- and defense-related proteins; lipid and membrane metabolism; amino acid metabolism; and uncharacterized proteins. Among these, the majority were associated with protein synthesis, processing, and degradation, followed by photosynthesis and carbohydrate metabolism (Figure 5A).

With respect to subcellular localization, the proteins were categorized into 13 groups (Figure 5B). Most proteins were localized to the chloroplast and chloroplast thylakoid membrane, followed by the cytoplasm, cell membrane, chloroplast stroma, cytosol, nucleus, apoplast, mitochondrion, ribosome, Golgi apparatus, and unknown locations.

The functional classification of identified proteins was carried out using gene ontology (http://geneontology.org/, accessed on 8 April 2026) based on the sorghum and maize genome annotation project database. Subcellular location was identified using SIB Bioinformatic resource portal (http://www.expasy.org/proteomics, accessed on 8 April 2026) with UniProtKB Complete proteome (http://www.uniprot.org/, accessed on 8 April 2026) annotation project databases.

## 4. Discussion

Our study focuses on the proteomic response of sorghum leaves to acute osmotic stress at a time point representing established physiological adjustment (24 h), rather than an early signaling phase. While we acknowledge that PEG treatment does not fully replicate the complex, gradual onset of soil drying or the associated physical and biological changes in the rhizosphere, it effectively isolates the cellular dehydration response. This allows us to dissect the molecular events triggered by water deficit after a full light-dark cycle, without confounding factors such as soil heterogeneity or differential root penetration. Moreover, our physiological measurements across multiple early time points (3, 6, and 9 h) provide a temporal context linking the initial osmotic adjustments to the proteomic changes observed at 24 h, offering insights into the transition from early responses to a more stable physiological state under drought stress.

### 4.1. Physiological Alteration in Leaves of Sorghum in Response to Drought Stress

Physiological analyses indicate that the drought-induced defense response was fully activated after 24 h of treatment, confirming this time point as optimal for proteomic investigation. Enhancing crop drought resistance requires a deeper understanding of the traits inherent in drought-tolerant sorghum plants to facilitate their integration into new varieties. This study was designed to elucidate the molecular mechanisms, specifically the proteomic alterations, in sorghum leaves under simulated drought stress. The integrated proteomic and physiological data provide valuable insights into the mechanisms underlying osmotic stress responses in sorghum plants.

### 4.2. Proteins Involved in Synthesis/Processing/Degradation

Six up-regulated protein spots—eukaryotic translation initiation factor 3 subunit F (eIF-3f, spot 2304), an uncharacterized protein (spot 3103), AAA domain-containing proteins (spots 3702 and 5006), a UVR domain-containing protein (spot 5805), and a PDZ domain-containing protein (spot 6403)—are involved in efficient protein synthesis and proteolysis. Protein synthesis plays a critical role in abiotic stress adaptation. Proteomic studies have shown that multiple components of the protein synthesis machinery alter their expression under drought stress, including ribosomal proteins, translation initiation and elongation factors, and chaperones [61]. As an abiotic stress, drought causes protein damage and/or degradation through oxidative injury or proteolytic activity. Thus, increased levels of proteins involved in synthesis and proteolysis are essential for repairing damaged proteins and restoring metabolic activity and growth in plant cells. Elevated abundance of such proteins has also been reported in sorghum under salt/drought and Cd/Cu stress [33,34,35,36,38], as well as in sugarcane and rice under salt/drought stress [61,62,63].

Five down-regulated protein spots were identified: a peptidase S1 domain-containing protein (spot 3005), a PPIase cyclophilin-type domain-containing protein (spot 0502), and three uncharacterized proteins (spots 2804, 2805, 5101). Among these, spots 2804 and 2805 belong to the HSP70 family and participate in protein refolding. Most heat shock proteins (HSPs) act as molecular chaperones that maintain protein stability and proper folding. HSP70 is a known stress-responsive protein induced by various abiotic stresses such as heat, cold, drought, salinity, and oxidative stress [7]. The reason for the observed reduction in these two chaperones under drought remains unclear; however, similar decreases in HSP70 have been reported in *Arabidopsis thaliana* [64] and *Agrostis stolonifera* [65] under salt stress. The uncharacterized protein spot 5101 is associated with ribosome assembly and protein translation, belonging to the ribosomal protein subunit (RPs) family. It forms part of the ribosomal stalk and facilitates ribosome interaction with GTP-bound translation factors. RPs are essential for protein synthesis and play important roles in metabolism, cell division, and growth [66]. The PPIase cyclophilin-type domain protein is implicated in protein folding, while the peptidase S1 domain protein is involved in proteolysis. Reduced abundance of these five proteins suggests impaired protein synthesis, processing, and turnover under drought stress.

The differential expression of various components of the translation machinery indicates the presence of a sophisticated regulatory mechanism controlling protein synthesis and proteolysis in response to drought.

### 4.3. Proteins Involved in Photosynthesis

Photosynthesis is a fundamental metabolic process in plants, generating carbon sources that are subsequently converted into energy-rich molecules via the Calvin cycle in chloroplasts [67]. This process is highly susceptible to environmental stress, and water deficit can cause detrimental effects and disruptions in photosynthetic activity. Four up-regulated drought-responsive proteins associated with photosynthesis were identified: two uncharacterized proteins (spots 1009, 2704), the Rubisco large subunit (spot 5105), and uroporphyrinogen decarboxylase (spot 5402). RuBisCO comprises eight identical large subunits that contain the catalytic site responsible for carbon fixation. Under stress conditions, the RuBisCO large subunit is often degraded into multiple fragments [68]. Additionally, the RuBisCO large subunit-binding protein beta subunit acts as a chaperone to maintain complex assembly and RuBisCO activity [61]. In this study, drought stress induced the expression of both the RuBisCO large chain and an uncharacterized protein (spot 2704). Alterations in RuBisCO large chain levels have also been reported in maize [69], rapeseed [70], *Populus cathayana* [71], *Arabidopsis* [64], and wheat [72]. The uncharacterized protein (spot 2704) was identified as a RuBisCO large subunit-binding protein beta subunit, whose increased abundance has been observed in *Glycine max* [73], *Arabidopsis* [23], and wheat [74]. The up-regulation of these two proteins under drought conditions likely supports photosynthetic efficiency and energy production. The putative uncharacterized protein (spot 1009) is associated with chlorophyll biosynthesis, while uroporphyrinogen decarboxylase (spot 5402) participates in heme biosynthesis—both being essential components of the Calvin cycle. The elevated levels of these four proteins suggest that the sorghum inbred line BT × 623 enhances carbon fixation and energy metabolism under drought stress.

The levels of two proteins—cytochrome b6-f complex iron-sulfur subunit (spots 5001, 6001) and transketolase_1 domain-containing protein (spots 4806, 5806)—were decreased in drought-treated sorghum leaves. The cytochrome b6-f complex iron-sulfur subunit is a chloroplast precursor, and a decrease in its abundance has previously been reported in *Aeluropus lagopoides* [75]. In this study, the down-regulation of this protein suggests impairment of the photosynthetic machinery under water deficit, likely through degradation of photosynthesis-related proteins. Transketolase is involved in the Calvin cycle, and its reduced level under stress has been observed in several plant species [75,76]. Our results align with prior proteomic studies, indicating that photosynthetic activity was compromised under drought conditions.

### 4.4. Proteins Involved in Carbohydrate Metabolism

Plants require the expression of carbohydrate metabolism-related proteins to sustain normal growth and development under stress conditions [77]. Carbohydrate metabolism is considered one of the most critical pathways regulating sugar synthesis, interconversion, and carbon partitioning in plants [36]. In this study, three proteins involved in carbohydrate metabolism were up-regulated under drought stress: cytoplasmic malate dehydrogenase (spot 6404), an uncharacterized protein (spot 6706), and glucose-1-phosphate adenylyltransferase (spot 7601). Cytoplasmic malate dehydrogenase is a key enzyme in the tricarboxylic acid (TCA) cycle. Its increased abundance has been reported in rice [78] and wheat [72] under salt stress. The uncharacterized protein (spot 6706) exhibits beta-glucosidase activity, which is associated with cyanogenic glycoside catabolism [79], and its elevated levels have been observed in sorghum seedlings under salinity stress [34]. Glucose-1-phosphate adenylyltransferase participates in starch biosynthesis, a component of glycan biosynthesis, and its increased expression in sorghum leaves under salt stress has been documented [80]. The up-regulation of these proteins in drought-stressed sorghum seedlings likely reflects the cellular demand for additional energy to cope with water deficit and repair damage. One protein related to carbohydrate metabolism, malic enzyme, showed reduced abundance under drought stress. Multiple spots (4701, 5005) were identified as malic enzyme with differing molecular weights and isoelectric points. We speculate that spot 5005 (increased intensity) may represent a degradation fragment of malic enzyme (spot 4701, decreased), though this hypothesis requires experimental validation. A moderate reduction in malic enzyme under drought stress has been previously reported in sorghum [33], consistent with our findings. This down-regulation may lead to decreased decarboxylation activity and reduced malate consumption.

### 4.5. Proteins Involved in Energy Metabolism

Energy metabolism underwent significant alterations under drought stress. In this study, two energy metabolism-related proteins showed abundance changes: the chloroplastic ATP synthase delta chain (spot 0101) was down-regulated, while an uncharacterized protein (spot 7801) was up-regulated. ATP synthase is a key enzyme for assessing photosynthetic capacity in plants [81]. It plays a central role in energy transduction in chloroplasts and mitochondria, and helps maintain chloroplast function during drought. The enzyme consists of two domains—F_0_ and F_1_—the latter comprising α, β, γ, δ, and ε subunits [68]. The expression of ATP synthase subunits is influenced by various stresses. Up-regulation of the α and β subunits has been observed in cotton [82] and sugarcane [83] under drought, and in rice under temperature stress [84,85], suggesting a positive correlation between ATP synthase expression and stress tolerance. In contrast, its down-regulation has been documented in wheat [86] and sunflower [81] under water deficit, as well as in sorghum under cadmium and copper stress [35,36]. Although differential expression of the ATP synthase δ subunit under drought has not been widely reported, our study detected its decreased abundance under simulated drought, consistent with observations in sugarcane under osmotic stress [62]. This reduction may lead to decreased ATP production under water deficit. The uncharacterized protein (spot 7801) is predicted to be the NADH-ubiquinone oxidoreductase 75 kDa subunit, a core component of mitochondrial complex I. This complex transfers electrons from NADH to the respiratory chain and participates in ATP metabolism. Its up-regulation has been reported in salt-tolerant rice seedlings [87], aligning with our results and underscoring the importance of sufficient energy supply for sorghum to cope with drought stress.

### 4.6. Proteins Involved in Transcriptional & Regulation

The transcriptional regulation of drought-responsive genes represents a key adaptive mechanism in plants under various stress conditions [29]. Proteomic analyses indicate that the abundance of transcription-related proteins is altered by drought stress and contributes significantly to drought resistance [61]. In this study, regulatory proteins including an uncharacterized protein (spot 0104, 6004) and maturase K (spot 6804) were significantly up-regulated under drought stress. The uncharacterized protein (spot 0104) is identified as an RNA helicase, a protein family involved in RNA unwinding, replication, and transcription. Previous studies have reported salt-induced up-regulation of RNA helicase in wheat [88] and Arabidopsis [64]. The uncharacterized protein (spot 6004) contains BAH and TFIIS domains, which are associated with chromatin binding and transcriptional regulation. Maturase K, an RNA processing and splicing-related protein, facilitates intron binding and modulates gene expression at the transcriptional level [35]. Its increased abundance has been observed under salt and copper stress [68,89]. These regulatory proteins likely function within transcriptional networks to coordinate diverse processes in response to drought.

Two additional proteins—an uncharacterized protein (spot 0209) and a guanylate cyclase domain-containing protein (spot 4201)—were down-regulated under water deficit (Table 3). The uncharacterized protein (spot 0209) belongs to a kinase family implicated in transcriptional regulation, while the guanylate cyclase domain-containing protein may participate in cyclic nucleotide biosynthesis. Their down-regulation may reflect an optimized transcriptional program under drought conditions.

### 4.7. Proteins Involved in Stress Response

Stress-related proteins play pivotal roles in plant adaptation to environmental stress [61,63,90]. In this study, two stress-associated proteins—a disease resistance protein (spot 0601) and catalase (CAT, spot 9603)—were down-regulated in sorghum seedling leaves under drought treatment, while a NAD(P)-binding domain-containing protein (spot 3208) was up-regulated under water deficit. Down-regulation of disease resistance protein has been previously observed in *Salicornia europaea* under salinity stress [91], consistent with our findings. CAT, localized predominantly in peroxisomes, catalyzes the decomposition of H_2_O_2_ into water and oxygen, thereby mitigating ROS accumulation under abiotic stress. Reduced CAT levels have been reported in citrus [76], cucumber [92], and barley [93] under salt stress. The down-regulation of CAT observed here suggests that drought-resistant sorghum may employ alternative ROS scavenging pathways under water deficit, a finding supported by our physiological data (Figure 3).

The up-regulated NAD(P)-binding domain-containing protein (spot 3208) is associated with defense responses. Increased expression of stress-responsive proteins under abiotic stress is commonly reported [7]. These results indicate that reprogramming of metabolic pathways represents a key mechanism of drought resistance in sorghum.

### 4.8. Proteins Involved in Lipid Membrane Metabolic

Two protein spots related to lipid metabolism—a PlsC domain-containing protein (spot 7104) and a PMR5N domain-containing protein (spot 8201)—exhibited decreased abundance under drought stress (Table 3). The PlsC domain-containing protein is implicated in cutin biosynthesis, while the PMR5N domain-containing protein functions as an integral membrane component. Lipid synthesis and efficient transport are essential for maintaining membrane structural homeostasis under stress conditions [61]. Thus, the down-regulation of these proteins suggests that drought stress disrupts lipid metabolism, triggering rapid membrane remodeling as part of cellular adaptation to drought.

### 4.9. Proteins Involved in Amino Acid Metabolic

During drought stress, proteins involved in amino acid metabolism, including an uncharacterized protein (spot 6704) and cysteine synthase (spot 7301), were up-regulated in sorghum leaves. Cysteine synthase is a key enzyme in cysteine biosynthesis and contributes to increased glutathione (GSH) levels—a central component of the GSH-ascorbate cycle that detoxifies hydrogen peroxide [94]. Up-regulation of cysteine synthase has been observed in rice seedlings under oxidative and salt stress [68], as well as in aluminum-treated rice roots [95]. In this study, elevated cysteine synthase expression may enhance sorghum drought tolerance by mitigating oxidative damage caused by reactive molecules. The uncharacterized protein (spot 6704) is a nitrate-responsive protein homologous to ferredoxin–nitrite reductase, which plays an essential role in nitrate assimilation and the nitrogen cycle. Water deficit can induce nitrate accumulation in plants [63], and elevated nitrate levels may become toxic, inhibiting metabolic processes. Ferredoxin–nitrite reductase alleviates this toxicity by reducing nitrite to NO or NH_3_, thereby supplying precursors for nitrogenous metabolites and supporting plant growth under stress. Up-regulation of this enzyme has been reported in salt-stressed wheat leaves and roots [72,88]. The overexpression of this ferredoxin–nitrite reductase homolog in our study underscores its significant role in sorghum’s adaptation to water deficit.

### 4.10. Uncharacterized Protein

In this study, four up-regulated protein spots (1109, 1206, 8303, 9105) were identified as putative uncharacterized proteins with unknown functions. Another up-regulated spot (5008) was characterized as a NAB domain-containing protein, which exhibits actin filament-binding activity, though its precise biological role remains unclear. As a C4 cereal, sorghum has had its full genome sequenced since 2009 [22], yet most of its gene products remain experimentally unvalidated and are annotated as hypothetical proteins in databases. Such proteins are computationally predicted from genomic data but lack experimental confirmation at the protein level [36]. The present findings align with recent sorghum proteomic studies [33,34,35,36,37,38,39,40,41,42,71,80].

The 43 confidently identified proteins corresponded to 38 unique gene products, as several proteins appeared in multiple spots on 2-DE gels with variations in molecular weight (Mr) and/or isoelectric point (pI). Examples include transketolase_1 domain-containing protein (spots 4806 and 5806), cytochrome b6-f complex iron-sulfur subunit (spots 5001 and 6001), and malic enzyme (spots 4701 and 5005). These differences may arise from post-translational modifications (PTMs)—such as glycosylation or phosphorylation—that alter protein charge or mass. Similar electrophoretic patterns have been widely reported [34,61,96,97,98]. The presence of a single protein in multiple gel locations may reflect PTMs, isoforms from multigene families, proteolytic fragments, products of alternative splicing, protein subunits, or modifications introduced during sample preparation.

## 5. Conclusions

Water deficit poses a global challenge that constrains crop quality and yield, with particularly severe impacts on resource-limited rural communities in developing regions. Sorghum is recognized for its inherent drought tolerance; however, its molecular-level responses remain insufficiently characterized. Investigating drought resistance mechanisms in C4 cereal crops may provide insights that could contribute to efforts aimed at improving plant drought resilience. This study presents an integrated physiological and proteomic analysis to explore the biological responses of sorghum to PEG-induced drought stress.

The physiological response of sorghum to drought stress observed in this study included enhanced water retention capacity and the mitigation of oxidative damage, possibly through the synergistic action of antioxidant enzymes such as SOD, POD, and PPO. Proteomic analysis of sorghum leaves revealed that changes in the abundance of multiple functional protein groups were associated with the response to drought stress. These included proteins involved in photosynthesis, carbohydrate and energy metabolism, transcriptional regulation, and reactive oxygen species (ROS) scavenging. In addition, alterations in proteins related to protein synthesis, processing, and proteolysis were identified, suggesting that these processes may contribute to the established phase of the drought stress response. Further studies will be required to validate the functional significance of these candidate proteins under true drought conditions.

## Figures and Tables

**Table 1:** Average number of protein spots of sorghum leaf revealed after 2DE of sorghum seedlings treated with 10% PEG-6000 at 24 h post treatment. Total numbers of up- or down-regulated spots were obtained after matching between control (CK) and treated with 10% PEG-6000 gels. Results are means of three independent replicates.

	Mock	PEG Treated
Total number of spots	708	613
Replicates		321
Up-regulated (Quality score > 80 and fold > 2.0)		36
Up-regulated (Quality score < 80 and fold > 2.0)		35
Down-regulated (Quality score > 80 and fold > 2.0)		18
Down-regulated (Quality score < 80 and fold > 2.0)		33
Other spots (fold < 2.0)		199

**Table 2:** Up-regulated protein spots of sorghum seedlings treated with PEG-6000 imitation drought stress at 24 h post treatment identified by PMF query. Criteria of selection for identification were a 2.0 folds change compared to the mock and a Mascot quality score of over 80. All values are means from three independent experiments.

Spot No (SSP) ^a^	Protein Function ^b^	Subcellular Location ^c^	Gene/Locus ^d^	Mock Average Qty	PEG-6000 Treated Average Qty	Fold Change ^e^
0104	ATP-dependent helicase activity, hydrolase activity, DNA binding, protein binding, ATP binding	nucleolus, cytoplasm	*Sb03g004420*	266.2 ± 19.2	533.9 ± 22.1	2.01
0206	Failed to identify	unknown	*N*	1087.2 ± 79.1	2173.1 ± 191.7	2.00
1009	carotenoid biosynthetic process, chlorophyll biosynthetic process unsaturated fatty acid biosynthetic process	chloroplast thylakoid membrane, thylakoid lumen	*Sb03g006310*	230.0 ± 33.6	467 ± 130.5	2.03
1109	acid phosphatase activity	unknown	*Sb09g005960*	118.8 ± 21.3	244.3 ± 51.4	2.06
1113	Failed to identify	unknown	*N*	244.3 ± 42.7	494.2 ± 77.3	2.02
1206	acid phosphatase activity	unknown	*Sb09g005960*	1610.4 ± 364.7	3269.5 ± 582.7	2.03
2005	Failed to identify	unknown	*N*	256.6 ± 13.3	518.9 ± 85.1	2.02
2104	Failed to identify	unknown	*N*	126.3 ± 17.7	255.6 ± 11.9	2.02
2304	Initiation factor, protein biosynthesis	Cytoplasm	*Sb09g026780*	378.4 ± 94.4	781.3 ± 124.3	2.05
2704	Protein import into mitochondrial intermembrane space, ‘de novo’ protein folding, protein refolding	Cytosol	*Sb10g001120*	318.1 ± 65.9	782.4 ± 134.3	2.46
3103	translation elongation factor activity, translational elongation, peptide biosynthetic process	Cytoplasm	*Sb08g002610*	603.5 ± 22.2	1259.9 ± 86.1	2.09
3208	Defense response	Chloroplast stroma, apoplast, thylakoid	*Sb09g001130*	419.7 ± 102.3	865.8 ± 206.5	2.06
3702	ATPase activity, metalloendopeptidase activity, proteolysis	chloroplast envelope, chloroplast thylakoid membrane	*Sb10g026830*	713.8 ± 21.6	1519.7 ± 242.4	2.13
4003	Failed to identify	unknown	*N*	185.4 ± 11.8	425.1 ± 32.1	2.29
4006	Failed to identify	unknown	*N*	184.4 ± 12.6	381.3 ± 74.8	2.07
4101	Failed to identify	unknown	*N*	256.5 ± 27.3	521.2 ± 56.4	2.03
5001	Electron transport, transport, oxidation-reduction process	chloroplast thylakoid membrane, chloroplast envelope, plasma membrane	*Sb09g020820*	637.0 ± 44.2	3275.9 ± 670.1	5.14
5005	malate metabolic process, pyruvate metabolic process	chloroplast	*Sb03g003230*	174.4 ± 37.0	400.5 ± 32.0	2.30
5006	ATPase activity, metalloendopeptidase activity, proteolysis	chloroplast envelope, chloroplast thylakoid membrane	*Sb10g026830*	237.0 ± 11.6	504.6 ± 32.9	2.13
5008	Actin filament binding	plasma membrane	*100812191*	157.7 ± 36.6	343.9 ± 78.7	2.18
5105	Photosynthesis, Calvin cycle, Carbon dioxide fixation, Photorespiration	chloroplast	*Sb01g038810*	492.2 ± 51.0	1080.0 ± 167.3	2.19
5402	protoporphyrinogen IX biosynthetic process, Porphyrin biosynthesis	chloroplast envelope, chloroplast stroma	*rbcl*	137.3 ± 8.6	302.8 ± 93.5	2.21
5805	Chaperone, protein metabolic process	chloroplast	*Sb06g014590*	87.4 ± 9.7	428.1 ± 53.2	4.90
6002	Failed to identify	unknown		698.6 ± 41.1	1489.0 ± 143.5	2.13
6004	BAH domain, chromatin binding, transcription, DNA-templated	nucleus	*Sb01g028380*	244.5 ± 40.8	495.0 ± 78.9	2.02
6403	protein binding, serine-type endopeptidase activity, proteolysis	chloroplast thylakoid, nucleus, thylakoid lumen	*Sb09g028940*	235.8 ± 35.9	700.1 ± 31.5	2.97
6404	carbohydrate metabolic process, malate metabolic process, tricarboxylic acid cycle, oxidation-reduction process	apoplast, plasma membrane, vacuole, chloroplast stroma	*Sb01g019280*	985.5 ± 51.3	1997.5 ± 103.2	2.03
6704	oxidoreductase activity, iron-sulfur cluster binding, heme binding, response to nitrate	mitochondrion, apoplast, chloroplast stroma	*Sb04g034160*	403.1 ± 42.3	808.8 ± 82.6	2.01
6706	beta-glucosidase activity carbohydrate metabolic process	Cytoplasm	*Sb08g007610*	806.8 ± 58.0	1644.1 ± 185.3	2.04
6804	mRNA processing, tRNA processing	chloroplast	*AFV09441*	71.9 ± 7.0	149.4 ± 14.9	2.08
7301	cysteine biosynthetic process from serine, Amino-acid biosynthesis	Cytoplasm	*NP_001105469*	434.3 ± 35.6	931.6 ± 17.4	2.15
7303	Failed to identify	unknown	*N*	2306.3 ± 199.7	5789.2 ± 434.9	2.51
7601	Starch biosynthesis, glycogen biosynthetic process	Chloroplast	*Sb01g008940*	249.6 ± 17.3	501.5 ± 23.1	2.01
7801	ATP synthesis coupled electron transport, response to oxidative stress	chloroplast, mitochondrial respiratory chain complex I	*Sb01g010210*	239.2 ± 16.9	546.8 ± 30.4	2.30
8303	uncharacterized protein	unknown	*Sb03g008870*	514.5 ± 62.7	1043.1 ± 73.4	2.03
9105	uncharacterized protein	unknown	*Selmodraft_442983*	241.1 ± 35.7	554.9 ± 66.3	2.30

Note: ^a^ SSP: Automatic allocation of protein serial number by PDQuest, spot no: spot number. ^b^ Function were identified using Phytozome v11.0 with Sorghum bicolor v3.1 (https://phytozome.jgi.doe.gov/pz/portal.html#!search?show=BLAST&method=Org_Sbicolor, accessed on 5 August 2024) genome annotation project databases and SIB Bioinformatic resource portal (http://www.expasy.org/proteomics, accessed on 5 August 2024) with UniProtKB Complete proteome (http://www.uniprot.org/, accessed on 8 April 2026) annotation project database. ^c^ Subcellular location was identified using SIB Bioinformatic resource portal (http://www.expasy.org/proteomics, accessed on 5 August 2024) with UniProtKB Complete proteome (http://www.uniprot.org/, accessed on 5 August 2024) annotation project databases. ^d^ Gene locus were identified using Phytozome v11.0 with Sorghum bicolor v3.1 (https://phytozome.jgi.doe.gov/pz/portal.html#!search?show=BLAST&method=Org_Sbicolor, accessed on 5 August 2024) genome annotation project databases and SIB Bioinformatic resource portal (http://www.expasy.org/proteomics, accessed on 5 August 2024) with UniProtKB Complete proteome (http://www.uniprot.org/, accessed on 8 April 2026) annotation project database. ^e^ Fold change = PEG treated average Qty/Mock average Qty. Qty: Normalized protein spot quantity.

**Table 3:** Down-regulated protein spots of sorghum seedlings treated with PEG-6000 imitate drought stress at 24 h post treatment identified by PMF query. Criteria of selection for identification were a 0.5 folds change compared to the mock and a Mascot quality score of over 80. All values are means from three independent experiments.

Spot No (SSP) ^a^	Protein Function ^b^	Subcellular Location ^c^	Gene/Locus ^d^	Mock Average Qty	PEG-6000 Treated Average Qty	Fold Change ^e^
0101	proton-transporting ATP synthase activity, rotational mechanism, ATP synthesis coupled proton transport	chloroplast thylakoid membrane.	*Sb04g027810*	2152.2 ± 117.5	742.8 ± 4.5	0.35
0209	protein kinase activity, protein phosphorylation, regulation of transcription, DNA-templated	Intracellular	*Sb08g018240*	500.6 ± 47.5	249.6 ± 13.8	0.50
0502	peptidyl-prolyl cis-trans isomerase activity, protein peptidyl-prolyl isomerization, protein folding	chloroplast stroma, chloroplast thylakoid membrane, thylakoid lumen	*Sb07g019320*	3192.0 ± 585.2	1562.2 ± 277.9	0.49
0601	signal transduction, defense response	cytosolic	*Bra022850*	351.3 ± 61.5	173.2 ± 17.0	0.49
2804	cellular response to unfolded protein, protein refolding	cytosol, nucleolus	*Sb01g039530*	1154.3 ± 116.9	491.2 ± 18.6	0.43
2805	cellular response to unfolded protein, protein refolding	cytoplasm	*Sb08g018750*	687.2 ± 134.3	269.0 ± 39.1	0.39
3005	Hydrolase, Protease, Serine protease, proteolysis	unknown	*Chlncdraft_57555*	1425.7 ± 104.3	665.7 ± 53.0	0.47
4113	Failed to identify	unknown	*N*	364.7 ± 60.7	138.7 ± 37.1	0.38
4201	intracellular signal transduction, cyclic nucleotide biosynthetic process	Intracellular	*Micpun_55932*	1307.3 ± 121.1	611.0 ± 87.3	0.47
4701	malate metabolic process, pyruvate metabolic process	chloroplast	*Sb03g003230*	1244.4 ± 198.6	310.9 ± 58.3	0.25
4806	Pentose-phosphate shunt	cytosol	*Sb10g002220*	3106.1 ± 234.4	1066.9 ± 62.8	0.34
5101	ribosome biogenesis, translation	large ribosomal subunit	*Sb01g038810*	966.1 ± 44.7	481.9 ± 41.0	0.5
5806	Pentose-phosphate shunt	cytosol	*Sb10g002220*	619.4 ± 47.9	294.4 ± 31.0	0.48
6001	electron transport, transport, oxidation-reduction process	chloroplast thylakoid membrane, chloroplast envelope, plasma membrane	*Sb09g020820*	3528.1 ± 185.8	1587.5 ± 348.6	0.45
7104	cutin biosynthetic process, dephosphorylation	Transmembrane, integral component of membrane, Membrane	*Sb01g008880*	715.0 ± 67.3	342.2 ± 19.7	0.48
7907	Failed to identify	unknown	*N*	252.1 ± 21.5	119.9 ± 9.4	0.48
8201	O-acetyltransferase activity	Golgi apparatus	*Sb07g022420*	2002.2 ± 202.8	856.5 ± 78.3	0.43
9603	hydrogen peroxide catabolic process, response to oxidative stress	Cytoplasm	*Sb04g001130*	3145.6 ± 509.9	827.8 ± 108.8	0.26

Note: ^a^ Automatic allocation of protein serial number by PDQuest. ^b^ Function were identified using Phytozome v11.0 with Sorghum bicolor v3.1 (https://phytozome.jgi.doe.gov/pz/portal.html#!search?show=BLAST&method=Org_Sbicolor, accessed on 8 April 2026) genome annotation project databases and SIB Bioinformatic resource portal (http://www.expasy.org/proteomics, accessed on 8 April 2026) with UniProtKB Complete proteome (http://www.uniprot.org/, accessed on 8 April 2026) annotation project database. ^c^ Subcellular location was identified using SIB Bioinformatic resource portal (http://www.expasy.org/proteomics, accessed on 8 April 2026) with UniProtKB Complete proteome (http://www.uniprot.org/, accessed on 8 April 2026) annotation project databases. ^d^ Gene locus were identified using Phytozome v11.0 with Sorghum bicolor v3.1 (https://phytozome.jgi.doe.gov/pz/portal.html#!search?show=BLAST&method=Org_Sbicolor, accessed on 8 April 2026) genome annotation project databases and SIB Bioinformatic resource portal (http://www.expasy.org/proteomics, accessed on 8 April 2026) with UniProtKB Complete proteome (http://www.uniprot.org/, accessed on 8 April 2026) annotation project database. ^e^ Fold change = PEG treated average Qty/Mock average Qty.

**Table 4:** Protein identification from 2-DE gels by Peptide Mass Fingerprint.

Spot No (SSP) ^a^	Protein Identification ^b^	Mascot Score ^c^	Matched Peptide	Sequence Coverage (%) ^d^	Estimated Mw(kDa)/PI ^e^	Experimental Mw(kDa)/PI ^f^	Gene/Locus ^g^	Accession No ^h^	Taxonomy ^i^
0101	ATP synthase delta chain, chloroplast precursor	120	8	44	26.7/4.84	22.3/4.20	*Sb04g027810*	XP_002454273	*Sorghum bicolor*
0104	Uncharacterized protein	89	11	23	37.2/7.23	25.3/4.75	*Sb03g004420*	XP_002455105	*Sorghum bicolor*
0209	Uncharacterized protein	77	12	18	86.6/6.58	28.7/4.69	*Sb08g018240*	XP_002442325	*Sorghum bicolor*
0502	PPIase cyclophilin-type domain-containing protein	204	26	57	46.7/4.83	41.7/4.58	*Sb07g019320*	XP_002444271	*Sorghum bicolor*
0601	Disease resistance protein	86	14	16	104.3/5.82	57.9/4.60	*Bra022850*	ACP30600	*Brassica rapa subsp. pekinensis*
1009	Uncharacterized protein	94	8	53	20.6/5.59	14.1/4.79	*Sb03g006310*	XP_002455210	*Sorghum bicolor*
1109	Uncharacterized protein	111	12	46	29.4/5.15	25.3/5.00	*Sb09g005960*	XP_002439412	*Sorghum bicolor*
1206	Uncharacterized protein	131	15	51	29.4/5.15	27.7/4.83	*Sb09g005960*	XP_002439412	*Sorghum bicolor*
2304	Eukaryotic translation initiation factor 3 subunit F (eIF-3f)	96	13	35	47.1/8.11	30.2/5.26	*Sb09g026780*	KXG22419	*Sorghum bicolor*
2704	Uncharacterized protein	281	35	59	61.9/5.47	62.7/5.17	*Sb10g001120*	XP_002437709	*Sorghum bicolor*
2804	Uncharacterized protein	163	23	39	71.4/5.09	77.1/5.10	*Sb01g039530*	XP_002468097	*Sorghum bicolor*
2805	Uncharacterized protein	186	28	49	71.4/5.13	78.8/5.15	*Sb08g018750*	XP_002442353	*Sorghum bicolor*
3005	Peptidase S1 domain-containing protein	81	12	24	67.1/9.81	16.8/5.31	*Chlncdraft_57555*	EFN56680	*Chlorella variabilis*
3103	Uncharacterized protein	84	11	34	26.5/8.5	21.8/5.37	*Sb08g002610*	XP_002442770	*Sorghum bicolor*
3208	NAD(P)-bd_dom domain-containing protein	134	11	42	31.9/6.99	29.6/5.27	*Sb09g001130*	XP_002439133	*Sorghum bicolor*
3702	AAA domain-containing protein	228	36	53	72.6/5.68	68.6/5.27	*Sb10g026830*	KXG20578	*Sorghum bicolor*
4201	Guanylate cyclase domain-containing protein	74	23	14	18.9/6.38	27.1/5.39	*Micpun_55932*	XP_002499691	*Micromonas* sp. *RCC299*
4701	Malic enzyme	151	26	39	69.9/6.23	60.7/5.55	*Sb03g003230*	XP_002455030	*Sorghum bicolor*
4806	Transletolase_1 domain-containing protein	203	29	49	69.1/5.41	80.1/5.51	*Sb10g002220*	KXG19207	*Sorghum bicolor*
5001	Cytochrome b6-f complex iron-sulfur subunit (EC: 7.1.1.6)	89	6	40	24.3/8.20	18.1/5.54	*Sb09g020820*	XP_002441121	*Sorghum bicolor*
5005	Malic enzyme	82	14	26	69.9/6.23	16.0/5.72	*Sb03g003230*	XP_002455030	*Sorghum bicolor*
5006	AAA domain-containing protein	218	33	47	72.6/5.68	12.5/5.65	*Sb10g026830*	KXG20578	*Sorghum bicolor*
5008	NAB domain-containing protein	77	27	14	20.8/5.29	18.3/5.71	*100812191*	XP_003556062	*Glycine max*
5101	Uncharacterized protein	134	16	63	24.4/8.73	22.8/5.53	*Sb01g038810*	XP_002465435	*Sorghum bicolor*
5105	Rubisco large subunit RuBisCO large chain family (EC = 4.1.1.39)	89	10	31	27.3/7.98	23.9/5.76	*rbcl*	AFC75624	*Premna microphylla*
5402	Uroporphyrinogen decarboxylase	119	14	37	43.5/6.98	38.6/5.76	*Sb01g036030*	XP_002467895	*Sorghum bicolor*
5805	UVR domain-containing protein	273	39	40	102.2/6.32	91.7/5.75	*Sb06g014590*	XP_002447724	*Sorghum bicolor*
5806	Transletolase_1 domain-containing protein	108	16	36	69.1/5.41	80.1/5.65	*Sb10g002220*	KXG19207	*Sorghum bicolor*
6001	Cytochrome b6-f complex iron-sulfur subunit (EC: 7.1.1.6)	94	10	43	24.3/8.20	18.2/5.71	*Sb09g020820*	XP_002441121	*Sorghum bicolor*
6004	Uncharacterized protein	73	15	16	14.8/6.71	16.7/5.67	*Sb01g028380*	KXG38864	*Sorghum bicolor*
6403	PDZ domain-containing protein	109	10	27	45.0/8.34	36.6/5.87	*Sb09g028940*	OQU78439	*Sorghum bicolor*
6404	Malate dehydrogenase (EC = 1.1.1.37)	106	17	46	35.8/5.76	37.9/5.87	*Sb01g019280*	XP_002467079	*Sorghum bicolor*
6704	Uncharacterized protein	165	30	41	66.4/6.33	62.9/5.81	*Sb04g034160*	XP_002454602	*Sorghum bicolor*
6706	Uncharacterized protein	149	14	42	35.4/6.42	26.0/6.10	*Sb08g007610*	XP_002443073	*Sorghum bicolor*
6804	Maturase K	85	10	23	34.2/9.50	77.7/5.91	*N/A*	AFV09441	*Drimia delagoensis*
7104	PlsC domain-containing protein	87	10	22	55.8/9.10	26.0/6.10	*Sb01g008880*	XP_002463916	*Sorghum bicolor*
7301	Cysteine synthase (EC = 2.5.1.47)	107	25	51	34.3/5.91	33.4/5.92	*Cys2*	NP_001105469	*Zea mays*
7601	Glucose-1-phosphate adenylyltransferase (EC = 2.7.7.27)	194	20	47	55.7/8.33	51.1/5.95	*Sb01g008940*	XP_002463921	*Sorghum bicolor*
7801	Uncharacterized protein	109	25	38	81.7/6.00	81.7/6.04	*Sb01g010210*	XP_002463995	*Sorghum bicolor*
8201	PMR5N domain-containing protein	76	7	25	39.2/8.59	25.8/6.43	*Sb07g022420*	XP_002444474	*Sorghum bicolor*
8303	Uncharacterized protein	77	8	34	31.6/6.06	31.6/6.34	*Sb03g008870*	XP_002457532	*Sorghum bicolor*
9105	Putative uncharacterized protein	80	13	39	40.0/5.30	24.6/6.89	*Selmodraft_442983*	XP_002976022	*Selaginella moellendorffii*
9603	Catalase (EC=1.11.1.6)	257	31	64	54.2/6.71	57.9/6.86	*Sb04g001130*	OQU84208	*Sorghum bicolor*

Note: ^a^ Automatic allocation of protein serial number by PDQuest. ^b,h,i^ Estimates based on NCBInr by Mascot procedure (in the Viridiplantae library). ^c^ Scores greater than 73 were considered significant (*p* < 0.05). ^d^ The highest matching value of sequence coverage. ^e^ Calculated by MS. Mw: Molecular weight. ^f^ Estimates based on 2D-gel data. PI: isoelectric point. ^g^ Gene locus were identified using Phytozome *v*11.0 with *Sorghum bicolor v3.1* (https://phytozome.jgi.doe.gov/pz/portal.html#!search?show=BLAST&method=Org_Sbicolor, accessed on 8 April 2026) genome annotation project databases and SIB Bioinformatic resource portal (http://www.expasy.org/proteomics, accessed on 8 April 2026) with UniProtKB Complete proteome (http://www.uniprot.org/, accessed on 8 April 2026) annotation project database.

**Table 5:** The 43 identified proteins in the leaves of sorghum seedlings within functional categories based on their biological functions and subcellular localization.

Categories	Classification Basis	Protein Spot Serial Number (Total 43)
Biological function	Transcription & regulation	0104 ↑, 0209 ↓, 4201 ↓, 6004 ↑, 6804 ↑
	Protein synthesis/processing/degradation	0502 ↓, 2304 ↑, 2804 ↓, 2805 ↓, 3005 ↓, 3103 ↑, 3702 ↑, 5006 ↑, 5101 ↓, 5805 ↑, 6403 ↑
	Photosynthesis	1009 ↑, 2704 ↑, 4806 ↓, 5001 ↑, 5105 ↑, 5402 ↑, 5806 ↓, 6001 ↓
	Energy metabolism	0101 ↓, 7801 ↑
	Carbohydrate metabolism	4701 ↓, 5005 ↑, 6404 ↑, 6706 ↑, 7601 ↑
	Defense responses	0601 ↓, 3208 ↑, 9603 ↓
	Lipid membrane metabolism	7104 ↓, 8201 ↓
	Amino acid biosynthesis	6704 ↑, 7301 ↑
	Uncharacterized protein	1109 ↑, 1206 ↑, 5008 ↑, 8303 ↑, 9105 ↑
Subcellular localization	Cytoplasm	0104 ↑, 2304 ↑, 2805 ↓, 6706 ↑, 7301 ↑, 9603 ↓, 3103 ↑
	Chloroplast	3702 ↑, 4701 ↓, 5001 ↑, 5005 ↑, 5006 ↑, 5105 ↑, 5402 ↑, 5805 ↑, 6404 ↑, 6804 ↑, 7601 ↑, 7801 ↑
	Chloroplast stroma	0502 ↓, 3208 ↑, 5402 ↑, 6404 ↑, 6704 ↑
	Chloroplast thylakoid membrane	0101 ↓, 0502 ↓, 1009 ↑, 3208 ↑, 3702 ↑, 5001 ↑, 5006 ↑, 6001 ↓, 6403 ↑
	Nucleus	0104 ↑, 2804 ↓, 6004 ↑, 6403 ↑
	Mitochondrion	6704 ↑, 7801 ↑
	Cell membrane	5001 ↑, 6001 ↓, 6404 ↑, 7104 ↓
	Ribosome	5101 ↓
	Intracellular	0209 ↓, 4201 ↓
	cytosol	0601 ↓, 2704 ↑, 2804 ↓, 4806 ↓, 5806 ↓
	Golgi apparatus	8201 ↓
	apoplast	3208 ↑, 6404 ↑, 6704 ↑
	Uncharacterized	1109 ↑, 1206 ↑, 3005 ↓, 8303 ↑, 9105 ↑

Note: The upward-pointing arrows indicate protein spots that were upregulated under PEG-induced drought stress; the downward-pointing arrows indicate protein spots that were downregulated under PEG-induced drought stress.

## Data Availability

The original contributions presented in this study are included in the article/Supplementary Material. Further inquiries can be directed to the corresponding author.

## Supplementary Materials

The following supporting information can be downloaded at: https://www.mdpi.com/article/10.3390/plants15081255/s1, Figure S1. Two-D gel analysis with proteins isolated from leaves of control sorghum seedling (A) or leaves of PEG-6000 treated sorghum seedlings (B) and harvested at 24 h post treatment.
