# Supplementary material for: Physiological and Proteomic Analysis of Sorghum Bicolor Seedling Leaves Reveals Molecular Responses to PEG-Induced Drought Stress
Source: Plants (Basel). 2026 Apr 18;15(8):1255. doi: 10.3390/plants15081255 (PMC13119943; doi:10.3390/plants15081255)

Figure S1. Two-D gel analysis with proteins isolated from leaves of control sorghum seedling (A) or leaves of PEG-6000 treated sorghum seedlings (B) and harvested at 24 hours post treatment. Arrows pointed to protein spots (Automatic allocation of protein serial number by PDQuest Software) with altered expression levels (fold change >2.0, quality score >80, $p < 0.05$) and were selected for MALDI-TOF-TOF analysis.

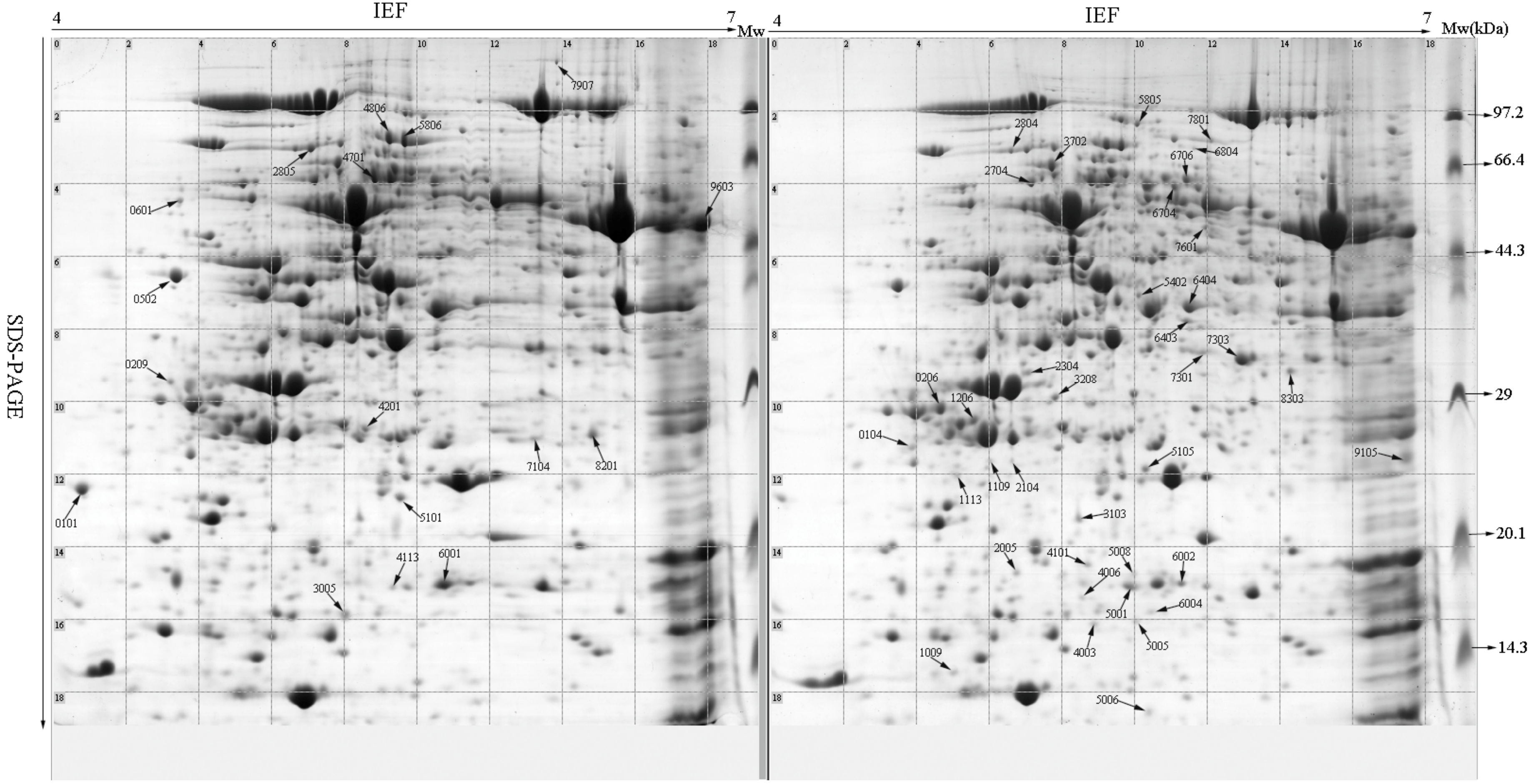

Supplement: Supplementary file 1 [file plants-15-01255-s001.zip › Figure S1.pdf]
